# Crude Oil Degrading Fingerprint and the Overexpression of Oxidase and Invasive Genes for *n*-hexadecane and Crude Oil Degradation in the *Acinetobacter pittii* H9-3 Strain

**DOI:** 10.3390/ijerph16020188

**Published:** 2019-01-10

**Authors:** Yang Wang, Qiuyu Wang, Limei Liu

**Affiliations:** 1 College of Life Science and Technology, Harbin Normal University, Harbin 150025, China; 2 College of Life Science, Northeast Forestry University, Harbin 150040, China; wqyll@sina.com; 3 School of Resources and Environmental Engineering, Guizhou Institute of Technology, Guizhou 550003, China; lmliu@126.com

**Keywords:** crude oil, GC-MS, fingerprints, biodegradation, *Acinetobacter pittii*

## Abstract

A crude oil-degrading bacterium named strain H9-3 was isolated from crude oil contaminated soil in the Northeastern area of China. Based on its morphological characteristics and 16S rDNA sequence analysis, strain H9-3 is affiliated to *Acinetobacter pittii* in the group of Gammaproteobacteria. The strain was efficient in removing 36.8% of the initial 10 g·L${}^{-1}$ of crude oil within 21 days. GC-MS was performed and a preference was shown for *n*-C10, *n*-C11, *i*-C14, *i*-C17, *i*-C34, *n*-C12, *n*-C13, *n*-C14, *n*-C27, *n*-C32 and *i*-C13, over *n*-C16, *n*-C18–C22, *n*-C24–*n*-C31, and *n*-C36. This can be regarded as the specific fingerprint for crude oil degradation by strain H9-3 of *Acinetobacter pittii*. In addition to crude oil, it was shown that soybean oil and phenols can be utilized as carbon sources by strain H9-3. It was also shown that aniline and α-naphthol cannot be utilized for growth, but they can be tolerated by strain H9-3. Methylbenzene was neither utilized nor tolerated by strain H9-3. Although *n*-hexadecane was not preferentially consumed by strain H9-3, during culture with crude oil, it could be utilized for growth when it is the sole carbon source. The degradation of some branched alkanes (*i*-C14, *i*-C17 and *i*-C34) and the preferential degradation of crude oil over phenols could be used as a reference for distinguishing *A. pittii* from *A. calcoaceticus*. The difference in gene expression was very significant and was induced by diverse carbon sources, as shown in the qRT-PCR results. The oxidation and adhesion events occurred at high frequency during alkane degration by *Acinetobacter pittii* strain H9-3 cells.

## 1. Introduction

Oil contamination is a worldwide problem, which is growing more serious with economic development. Its effects are long lasting and remediation is challenging [1]. Bioremediation, which has the advantages of good effect, low cost, little impact on the environment, no secondary pollution, and a wide application range, is a better way to control petroleum hydrocarbon pollution [2]. When adding bacteria to the contaminated soil, the process of undergoing biological treatment is referred to as a bioaugmented treatment system [3]. It provides higher efficiency and safety in the process of the bioremediation of oil-contaminated soil [4].

The ability to oxidise alkanes is widespread among microorganisms [5,6]. Many microorganisms can utilize petroleum hydrocarbons [7,8,9,10,11,12,13]. *Acinetobacter* sp. strains are most often found in contaminated habitats [3] and act as the best degraders for the bioremediation of soil polluted by crude oil [14]. The marine crude oil-degrading bacterium *Acinetobacter* sp. strains have been cultured on an industrial scale [14]. Study on the multiple catabolic capacity of 31 isolates belonging to the genus *Acinetobacter* has shown multiple degradation of hydrocarbons, dibenzothiophene, fluorene, dibenzofuran, benzyl sulfide, and sodium benzoate [15]. As a member of *Acinetobacter* sp., *A. calcoaceticus*, strains exploit a diverse range of hydrocarbons as sources of energy and carbon, and this has been substantiated by prior researches. The first report, in 1995, showed that *A. calcoaceticus* MM can degrade and emulsify heating oil [15]. The straight-chain of *n*-alkanes in the fuel can be degraded essentially, but the branched hydrocarbons, pristane, and phytane cannot be [16]. It is widely accepted that *A. calcoaceticus* strains have more potential application in bioremediation [17]. The isolation from an oil-contaminated sites in the Persian Gulf and the Caspian confirmed it to be the most effective strain for the degradation of crude oil [18]. Besides alkanes, phenols can also be utilized by *A. calcoaceticus* strains as the sole source of carbon and energy [19,20]. *Acinetobacter calcoaceticus* PHEA-2 isolated from industrial wastewater showed a strong ability to degrade phenols [21,22]. The genus *Acinetobacter* currently comprises 56 validly named species (April 2018) [23]. *A. calcoaceticus* and *A. pittii* are closely related species [24], so by use of MLST (Multi Locus Sequence Typing, a nucleotide sequence based approach for the unambiguous characterisation of isolates of bacteria and other organisms via the internet) and rpoB gene sequencing analysis *A. calcoaceticus* PHEA-2 was renamed *Acinetobacter pittii* PHEA-2 [25]. *Acinetobacter pittii* sp. was first reported as a new genomic species by Nemec et al. [26] in 2011. Much attention has been drawn to the *A. Calcoaceticus* strain as a microcomposer to many contaminants, such as chlorpyrifos, fipronil, Ochratoxin, hexachloroplatinic acid, caprolactam, herbicide propanil, and furazolidone [27]. Meanwhile, in some studies, *A. calcoaceticus* strains showed strong tolerance and resistance to antibiotics and Cu${}^{2+}$. All of these events made us more interested in the “dietary bias” of *Acinetobacter* sp. strains in regard to explaining the metabolic pathways and gene functions for alkane biodegradation.

Furthermore, the establishment of an oxidase-based catalytic degradation system in vitro is a promising method for environmental remediation. Oxygenase is often derived from the fermentation of microorganisms, so it is meaningful to study which substrates specific strains can act on.

In this study, a bacterial strain labeled as H9-3 was isolated from crude oil-contaminated soil in the Northeastern part of China. The strain was identified as *Acinetobacter pittii* by sequence analysis of 16S rDNA genes. The degradation rate of crude oil by strain H9-3 was then determined. In addition, degradation fingerprints for crude oil were determined by GC-MS analysis. Interestingly, and different from previous reports, we found a serious “dietary bias” in the process of alkane degradation by *A. pittii* H9-3. Furthermore, the expression levels of several genes related to alkane degradation induced by different carbon sources were compared. This study proves that oxidation and adhesion events occur at a high frequency in the crude oil degradation pathway.

## 2. Materials and Methods

### 2.1. Enrichment, Isolation, and Identification of Crude Oil-Degrading Bacteria

Crude oil-contaminated soil samples were collected from the shallow soil around an oil well located in Daqing oilfield that was exploited in 1990s. The samples were inoculated into flasks containing MSM (Mineral Salt Medium) with crude oil (10 g·L${}^{-1}$) for the enrichment culture. The subculture was performed in triplicate. The final enriched media was diluted serially and spread on LB (Luria-Bertani culture medium) agar plates supplemented with chicken blood (20 mL·L${}^{-1}$). The plates were incubated at 30 ${}^{\circ }$C, and single colonies with a hemolytic circle were selected and streaked on new plates. The resulting isolates were stored at 4 ${}^{\circ }$C until further study.

Bacterial cell morphology was examined by transmission electron microscopy (Hitachi, Tokyo, Japan). For characterization by 16S rDNA, the genomic DNA of strain H9-3 was extracted and used as a template to amplify bacterial 16S rDNA with universal primers 25F (5’-AGAGTTTGATCATGGCTCAG-3’) and 1421R (5’-TACGGTTACCTTGTTACGACTT-3’) on a Mastercycler gradient thermocycler (Eppendorf, Hamburg, Germany). The PCR products were sequenced, and the sequences were compared with bacterial 16S rDNA sequences in EzBioCloud using the Basic Local Alignment Search Tool (IDENTIFY) program [28]. Neighbor-joining phylogenetic trees were constructed using the Molecular Evolutionary Genetics Analysis (MEGA) program. The reliability of the phylogenetic reconstructions was estimated through bootstrap analysis (1000 replicates). The H9-3 strains were preserved in the Genetics Laboratory of Northeast Forestry University.

### 2.2. Crude Oil Degradation by Strain H9-3

Strain H9-3 cultured liquid (2 mL, OD${}_{600}$ = 0.6) was inoculated into 100 mL CMSM (MSM with 10 g·L${}^{-1}$ crude oil) in flasks sealed with a filter to test the degradation rate of crude oil. The crude oil produced in Daqing oilfield is a medium paraffin-based crude oil with a high wax content, low sulfur content, high viscosity, and high solidification point. The properties of Daqing crude oil are as follows: density (ρ at 20 ${}^{\circ }$C, g/cm${}^{3}$) 0.86, viscosity (mm${}^{2}$/S) 22.2, wax content (*W*%) 26.2, acidity value 0.08, C (*W*%) 85.74, H (*W*%) 13.31, S (*W*%) 0.11, and N (ppm) 1586 (from Sinopec crude oil analysis report, June 14, 2011). The degradation test was incubated at 150 rpm for 21 days with growth occurring under conditions of 30 ${}^{\circ }$C and pH 8.0. At last, samples were collected periodically to measure the biomass and crude oil degradation. The biomass contents were monitored spectrophotometrically by measuring the absorbance at 600 nm. The crude oil degradation rate (η, %) was calculated as η = (*W*${}_{ol}$ − *W*)/*W*${}_{ol}$, where *W*${}_{ol}$ and *W* are the weights (g) of crude oil in the original liquid (OL) and the remedial liquid, respectively. The efficiency of crude oil degradation was determined by the Meteorological Mass Spectrometer technique. Duplicate samples were mixed and divided into two groups based on whether they contained strain H9-3. Then, crude oil was extracted from those mixed liquids and injected into an HP6890N (Agilent Technologies, Santa Clara, CA, USA) plus GC equipped with a Phenomenex Zebron ZB-5 MS Capillary column (30 m × 0.25 mm i.d. × 0.25 μm film thickness) (Supelco, Bellefonte, PA, USA). As a detector, we used an HP5973 mass selective detector (mass range: 15–500 amu; scan rate: 1.9 scans/s; EM voltage: 1435), and helium at 1 mL·min${}^{-1}$ was used as the carrier gas. The injection port was split at 280 ${}^{\circ }$C. The injection volume was 1 μL. The detector was maintained at 230 ${}^{\circ }$C. The oven was maintained at 40 ${}^{\circ }$C for 4 min, and then the temperature was increased to 250 ${}^{\circ }$C (10 ${}^{\circ }$C/min), and this temperature was maintained for 10 min. The ionization method used was electron impact ionization (EI). The ionsource was operated in the electron ionization (EI) mode at 70 eV. The resolution of the mass detector is 0.1 m/z. All of the analyses were performed in triplicate (relative standard deviation [RSD] 0.02%). All peaks were identified from their mass spectra by comparison with spectral NIST 02 MS libraries. The alkanes in samples were qualitatively analyzed by GC-MS in terms of retention time and characteristic ions, and they were quantified by the internal standard method. C${}_{24}$D${}_{50}$ was chosen as the internal standard because the properties were stable and no reacting with alkanes in the sample. Quantitative methods, such as those described by Wang et al. [29], were summarized as follows:

Drawing Standard Curves of Alkanes: The standard curve of alkane concentration was prepared by using standard solution of mixed alkane (C7-C40 Saturated Alkane Mixture, Sepulco Company, USA) and internal standard substance (C${}_{24}$D${}_{50}$, Chem Service Company, USA). The specific method is to dilute the standard solution of mixed alkane, take 1 mL of each five gradient concentration, then add 100 μL of internal standard substance, sample injection and GC-MS analysis, plot the peak area ratio concentration ratio, and get the standard curve.

Quantitative analysis of samples: The samples were dehydrated by anhydrous sodium sulfate and then separated by silica gel column chromatography. After nitrogen blowing, the sample was concentrated and the volume was 1 mL, then adding 100 μL of internal standard substance, sampling and GC-MS analysis. The peak area ratio of the sample to the internal standard substance was obtained, and then by comparing to the standard curve, the concentration of alkanes in the sample could be obtained.

### 2.3. Utilization of Other Carbon Sources by A. pittii H9-3

To test the growth conditions of strain H9-3 using different hydrocarbons as sole carbon sources, 1 mL seed liquid of strain H9-3 was inoculated in 100 mL of MS medium supplemented with crude oil, soybean oil, α-naphthol, aniline, phenols, and methylbenzene in 150 mL Erlenmeyer flasks. The final concentrations in the flasks were 1% (m/v) for crude oil, 5% (v/v) for soybean oil, 0.01% (v/v) for α-naphthol, 0.1% (w/v) for aniline, 0.1% for phenols (w/v), and 0.1% for methylbenzene (v/v). Two controls, one containing strain H9-3 cells and no hydrocarbons and another containing hydrocarbons and no H9-3 cells, were used for calculation of the background baseline of growth and degradation. All cultures were incubated in the dark at 30 ${}^{\circ }$C while shaking at 150 rpm.

### 2.4. Differential Expression of Alkane-Degrading Genes in Strain H9-3 Cells under Different Carbon Source Culture Conditions

In order to investigate the differences in gene expression related to alkane degradation induced by different carbon sources, strain H9-3 was cultured in MSM with the additive of either crude oil or *n*-hexadecane as the sole carbon source, and meanwhile, LB medium was used as the H9-3 control. The cells of strain H9-3 were collected by centrifuge at a rotary speed of 8000 rpm once the OD${}_{600}$ value of the culture liquid reached 0.6. Then, total RNA was extracted from the collected cells to be templates of the RT-PCR reaction (TaKaRa PrimeScript 1st Strand cDNA Synthesis Kit) to obtain the cDNA to be used as the template for qRT-PCR reactions. The primer used in qRT-PCR was designed according to the gene sequence concerned with alkane degradation. The gene sequences were obtained from the GenBank of the NCBI. All primer sequences (Table 1) were synthesized by Sangon Biotechnology Co., Ltd. (Shanghai, China).

In order to determine the validity of the primers, PCR amplification reactions were first performed. After suitable PCR primers had been ensured, real-time quantitative PCR (qRT-PCR) was used to investigate the differences in gene expression. The q-PCR reaction used a SYBR fluorescent dye kit (GoTaq^®^ 2-Step RT-qPCR System), and the q-PCR program was the following: 95 ${}^{\circ }$C for 1.5 min; 40 cycles composed of 95 ${}^{\circ }$C for 30 s, 58 ${}^{\circ }$C for 30 s, 72 ${}^{\circ }$C for 30 s, and 79 ${}^{\circ }$C for 1 min; then 72 ${}^{\circ }$C for 10 min and a 10 ${}^{\circ }$C hold. For data redaction and statistical analysis, we used the $2^{-{\Delta \Delta C}_{T}}$ methods of Livak and Schmittgen [31].

## 3. Results

### 3.1. Isolation, Characterization, and Identification of Crude Oil-Degrading Strains

**Figure 1:**
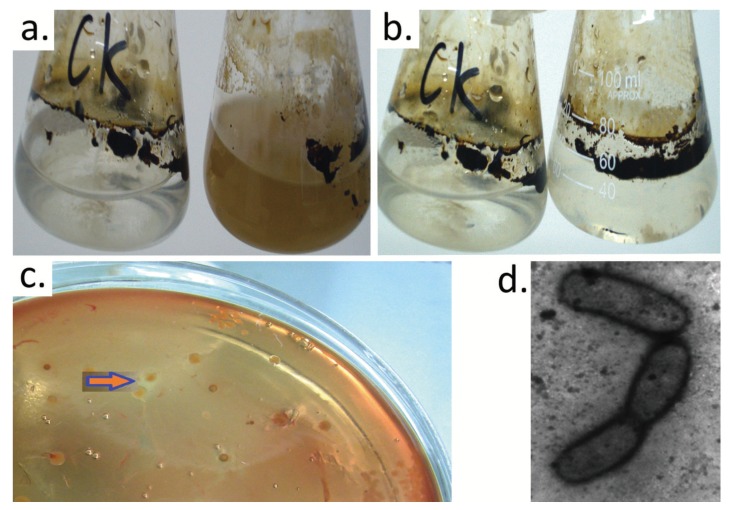
Enrichment, characterization, and identification of crude oil-degrading bacteria: (**a**) good enrichment (The left is the control (CK) that was the mineral culture medium containing crude oil without adding oil contaminated soil and the right is the sample); (**b**) bad enrichment (the left is CK and the right is the sample); (**c**) hemolytic rings of the colony for strain H9-3; (**d**) transmission electron micrograph of *Acinetobacter pittii* H9-3 (60,000×).

After three weeks of enrichment culture (Figure 1a,b), a total of 20 isolates were obtained on the LB agar blood plates with 100 μL of a 10${}^{5}$–10${}^{6}$ fold dilution of enrichment culture. The monocolony that exhibited a hemolytic ring (Figure 1c) and fast growth in crude oil-containing media was chosen and named H9-3. This was applied in the following study. The H9-3 strain was short rod-shaped bacteria with a cell size of approximately 1.42 μM in length and 0.65 μM in diameter under the microscope (Figure 1d).

**Figure 2:**
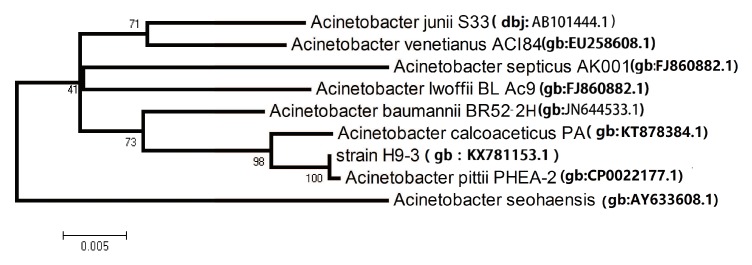
Phylogenetic tree presenting the position of strain H9-3.

The 16S rRNA gene of strain H9-3 was sequenced (GenBank accession numbers: KX781153.1) and used to construct a phylogenetic tree for further analysis. The partial sequence of the 16S rRNA gene was a continuous stretch with 1421 bp. The 16S rRNA sequence was input into EzBioCloud which provides proven similarity-based searches against quality-controlled databases of 16S rRNA sequences. Strain H9-3 was identified and affiliated with *Acinetobacter pittii* on EzBioCloud’s Identify service [28]. The phylogenetic analysis (1400 unambiguous bases aligned) classified strain H9-3 into the *Acinetobacter* genera, which belongs to the Gammaproteobacteria sub-phylum. The similarities between the strain H9-3 sequence and the other deposited bacterial sequences were caculated, and the H9-3 sequence showed 100% similarity to PHEA-2. By means of the neighbor-joining methods, a phylogenetic tree was constructed which indicated that the closest relative of strain H9-3 is *Acinetobacter pittii* PHEA-2 (Figure 2).

### 3.2. Degradation Components for Crude Oil by Acinetobacter pittii H9-3

**Figure 3:**
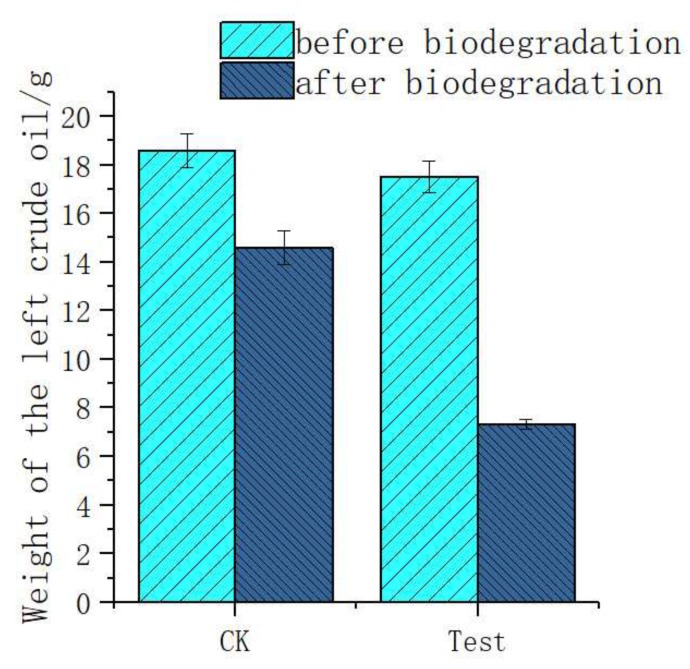
Comparison of the residual oil content before and after degradation.

Before the biodegradation experiment, there was an average of 18.57 g crude oil in the CK (control check) flasks not containing the H9-3 strain and 17.5 g crude oil in the test flasks containing the H9-3 strain. After biodegradation for 21 days, there was an average of 14.66 g of crude oil left in the CK flasks, while there was only 7.33 g of crude oil left in the test flasks. So, the degradation rate of crude oil by the H9-3 bacteria strain was 36.8%. A comparison of the residual oil content before and after degradation is shown in Figure 3.

**Figure 4:**
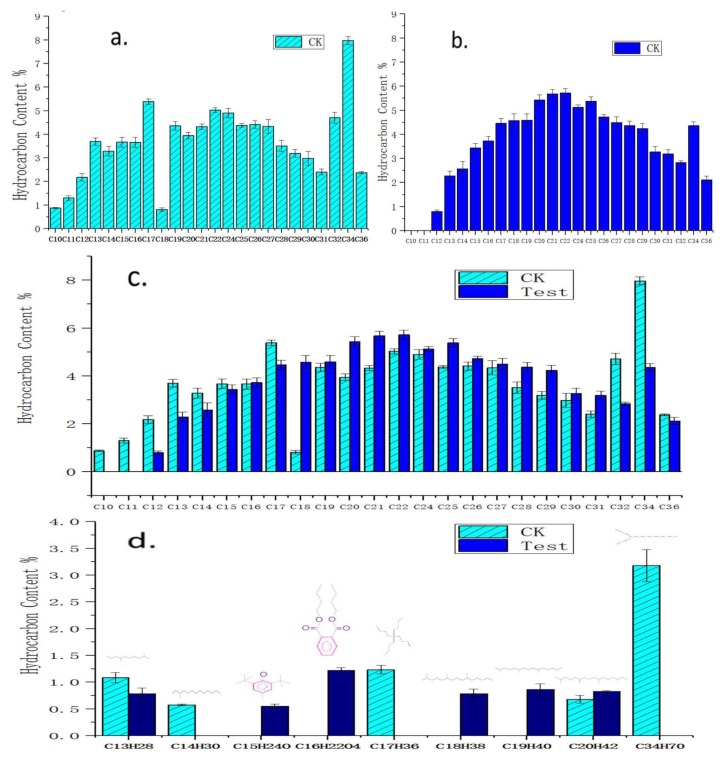
Compound contents in residual crude oil after biodegradation by the H9-3 strain. CK: Crude oil incubated for 21 days with no additive; Test: Crude oil incubated for 21 days with additives *A. pittii* H9-3). (**a**) The content of hydrocarbons in crude oil before biodegradation (day 0); (**b**) the content of hydrocarbons in crude oil after 21 days of biodegradation by strain H9-3; (**c**) *n*-alkane in crude oil after biodergadation; (**d**) *i*-alkane and oxygenated compounds in crude oil after biodegradation.

The components of crude oil before and after degradation were analyzed respectively by using gas chromatography-mass spectrometry (GC-MS). The main constituents of both samples were saturated, and the number and percentage of alkanes varied. By using the GC-MS method, 35 kinds of alkane were detected in crude oil medium without the H9-3 strain (Figure 4a), while 31 kinds of alkane were detected after culturing with the H9-3 strain for 21 days (Figure 4b).

The test results showed that within the scope of *n*-alkanes, the C10 and C11 compounds completely disappeared and the C12, C13, C14, C17, and C32 compounds decreased obviously in crude oil after biodegradation by strain H9-3 for 21 days. The contents of the C15 and C34 compounds remained unchanged, while increases in the relative amounts of the C16, C18–C22, C24–C31, and C36 fractions were observed, as shown in Figure 4c. It was confirmed there was a specific fingerprint when the H9-3 strain was living in crude oil, namely, a preference for C10, C11, C12, C13, C14, C17, and C32 *n*-alkanes rather than C16, C18–C22, C24–C31, and C36. This could be regarded as the identification marker of *Acinetobacter pittii* H9-3.

After biodegradation for 21 days, some other *i*-alkanes and oxygenated compounds in addition to *n*-alkanes were found to have undergone changes in content. For example, some compounds (*i*-C14H30, *i*-C17H36, *i*-C34H70) were consumed completely, while some compounds (C${}_{15}$H${}_{24}$O, C${}_{16}$H${}_{22}$O${}_{4}$, *i*-C18H38, *i*-C19H40) were newly formed (Figure 4c).

Two oxygenated compounds (C${}_{15}$H${}_{24}$O and C${}_{16}$H${}_{22}$O${}_{4}$) with a phenyl group (Figure 4d) were found. The m/z values leading to the determination of the two oxygenated compounds were as follows: butylated hydroxytoluene (BHT; Mz${}^{+}$220,145,177); dibutyl phthalate (Mz${}^{+}$: 149,223,205,121). The assignment of these two molecules was questionable because the resolution of the detector was not good enough to guarantee the identification of these two compounds, so validation of these compounds is needed in the future. These two compounds might have also been from contaminants in the experiment.

### 3.3. Growth of A. pittii H9-3 in Different Carbon Sources

**Figure 5:**
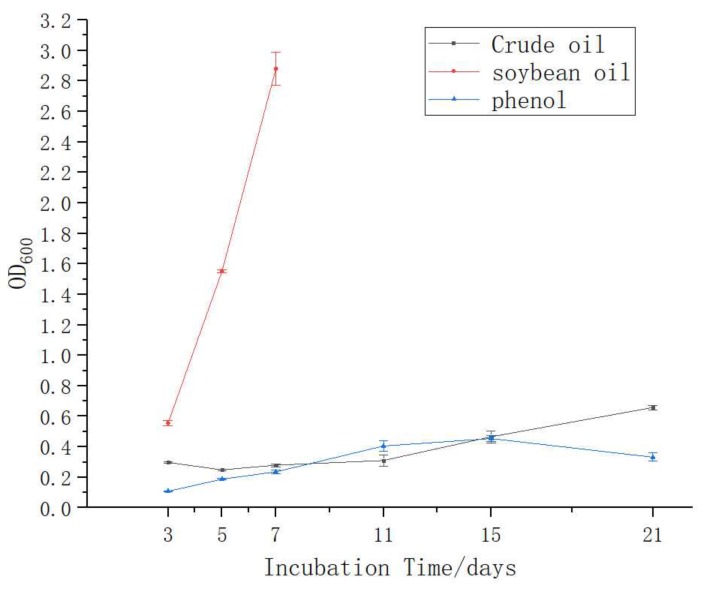
Growth of the H9-3 strain in different carbon sources for 21 days.

When crude oil, soybean oil, phenols, α-naphthol, and aniline were used as the sole carbon sources for the growth of the H9-3 strain that was observed by use of the determination of the OD600 value of the bacterial culture medium, the results showed that the H9-3 strain grew obviously and rapidly with soybean oil, while it grew a bit slower in crude oil and phenols. It could not use α-naphthol and aniline for growth. Growth was not observed when methylbenzene was the sole carbon source (Table 2). The H9-3 strain seemed to be more willing to degrade crude oil (1%) than phenols (0.1%) (Figure 5).

### 3.4. The Different Expression of Alkane-Degrading Genes in the H9-3 Strain Cultured with Different Carbon Sources

**Figure 6:**
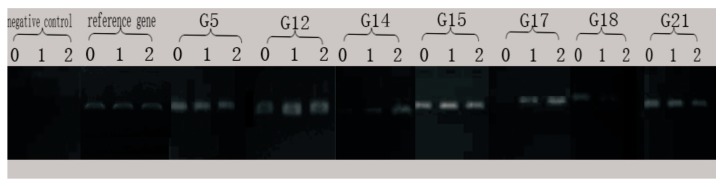
PCR electrophoresis of differentially expressed genes. Notes: 0: ck (LB); 1: test sample 1 (*n*-C16); 2: test sample 2 (crude oil).

The results of the electrophoresis of PCR products for alkane-degrading genes in the H9-3 strain cultured in different conditions are shown in Figure 6. According to the electrophoresis map, the bands of PCR amplification were single, which indicates that the primers had good specificity. It is worth noting that the differential expression of the G14, G17, and G18 genes in the H9-3 strain under different carbon source cultures can be seen from the PCR electrophoresis map.

**Figure 7:**
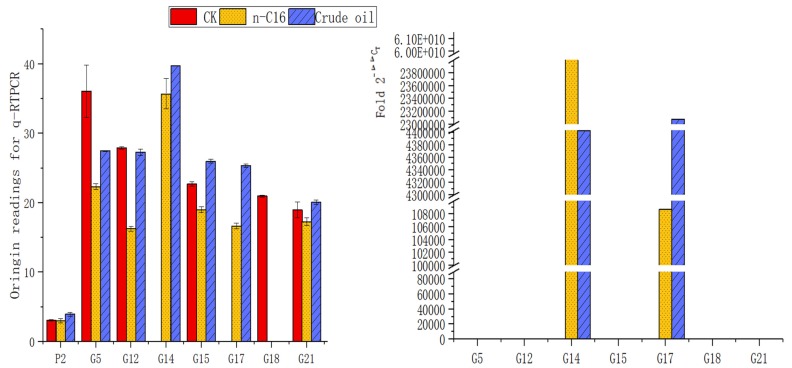
Comparison of gene expression induced by different carbon sources. **Left**: Original readings of qRT-PCR amplification for alkane-degrading genes: the reading for G14 and G17 in CK is “0”; the reading for G18 in *n*-C16 and crude oil is “0”. **Right**: Fold $2^{-{\Delta \Delta C}_{T}}$ for different expression levels of alkane degrading genes.

The specificity of PCR amplification was satisfying, and some specific bands were detected in 2–4 cycles, indicating that the specificity was very strong. The detection signal data accumulated value represents the level of gene expression, and the original readings are shown in Figure 7 (left). It can be seen that the reference gene expressed stability in all samples, respectively 3.08, 3.02, and 3.09, while alkane-degrading genes were expressed differently in control samples (CK) versus test samples (*n*-C16 and crude oil).

By setting the Ct value at 0.01 and assuming that the expression level of each gene was 1 in the control, the relative expression levels of the alkane-degrading genes were calculated and are shown in Figure 7 (right). Obviously, the overexpression of some genes, such as G14 and G17, was very significantly induced by crude oil or *n*-hexadecane. The expression level of G14 (oxidase genes) induced by *n*-hexadecane was 5.98 × 10${}^{10}$ times higher and that induced by crude oil was 4.4 × 10${}^{6}$ times higher than that of the control, while the expression level of G17 (invasive genes) induced by *n*-hexadecane was 1.08 × 10${}^{5}$ times higher and that induced by crude oil was 2.31 × 10${}^{7}$ times higher than that of the control. The G14 and G17 genes were reported to be an oxidase gene and an invasive gene, respectively according to blast (n) in NCBI.

Figure 7 (left) shows that some genes, such as G14 (oxidase), G15 (benzoate 1,2-oxygenase ion transfer complex), and G17 (adhesin and invasin), were up-regulated by crude oil or *n*-hexadecane in the H9-3 strain, while some genes, such as G5 (naphthalene degradation gene), G18 (bacteria ferritin-associated ferredoxin) and G21 (benzoate membrane transport protein), were down-regulated.

## 4. Discussion

GC-MS was performed to test crude oil components as additives incubated with *A. pittii* H9-3 for 21 days. It was indicated that the *n*-C10, *n*-C11 and *i*-C14H30, *i*-C17H36, *i*-C34H70 compounds decomposed completely while disappearing into the crude oil. At the same time, the reduction of the *n*-C12, *n*-C13, *n*-C14, *n*-C17, *n*-C32, and *i*-C13 compounds was also evident. However, the biodegradation of the *n*-C15, *n*-C34, *n*-C16, *n*-C18–*n*-C22, *n*-C24, *n*-C31, *n*-C36 and *i*-C20 compounds through degradation by the H9-3 strain was not obvious. Some branched alkanes, such as *i*-C14H30, *i*-C17H36 and *i*-C34H70, were utilized by the H9-3 strain as a preferential carbon source, but pristane and pentadecane were accumulated. That meant the H9-3 strain was the preference for *n*-C10, *n*-C11, *i*-C14, *i*-C17, *i*-C34, *n*-C12, *n*-C13, *n*-C14, *n*-C17, *n*-C32, and *i*-C13, rather than for *n*-C16, *n*-C18–C22, *n*-C24–*n*-C31,, and *n*-C36. This can be regarded as the specific fingerprint for crude oil degradation by the *Acinetobacter pittii* H9-3 strain. This fingerprint for the degradation of crude oil could assist in the classification of *Acinetobacter pittii* strains.

There was no evidence of *n*-hexadecane degradation when crude oil was used as a sole carbon source, but it was utilized for growth when *n*-hexadecane was the sole carbon source by the H9-3 strain. Due to the difference in oxygenase systems between the process of consuming *n*-hexadecane and crude oil, it was demonstrated that *n*-hexadecane and crude oil have different metabolic pathways.

*A. calcoaceticus* MMS underwent catabolism in *n*-alkane components but not in the branched hydrocarbons in the fuel [16]. Different from the MMS strain, both short chain *n*-alkanes and branched alkanes (*i*-C14H30, *i*-C17H36, *i*-C34H70) of crude oil were assimilated by the *A. pittii* H9-3 strain. This supports a difference in classification between the *A. pittii* and *A. calcoaceticus* strains.

The accumulation of pristane was shown to hinder a further increase in the degradation rate for the disposal of crude oil in previous studies [16]. In this study, we found that not only did pristane accumulate during crude oil degradation by the H9-3 strain, but also 2,6,10-trimethyl-pentadecane, which has a very similar structure. This proves that there is no benefit to bacterial growth from the production and accumulation of homologues with pristane analogues.

*A. Calcoaceticus* strains can utilize phenols as the sole source of carbon and energy [19,20]. The *A. Calcoaceticus* strain PA was efficient in removing 91.6% of the initial 800 mg·L${}^{-1}$ of phenols within 48 h, and had a phenol concentration tolerance as high as 1700 mg·L${}^{-1}$. We found that the H9-3 strain could also utilize the initial 1000 mg·L${}^{-1}$ of phenols as the sole source of carbon. Both *A. Calcoaceticus* and *A. pittii* have the ability to degrade phenols, which could be their similarity.

It has been reported that *Acinetobacter* sp. has both the phenol hydroxylase gene and the alkane monooxygenase gene. They can simultaneously degrade phenols and *n*-hexadecane for growth, but prefer phenols over *n*-hexadecane [32]. In our study, the *A. pittii* H9-3 strain seemed to be more willing to degrade crude oil than phenols. This is probably the evolutionary difference between *A. Calcoaceticus* and *A. pittii*.

Due to the induction of G14 and G17 overexpression by crude oil or *n*-hexadecane, there is reason to believe that oxidation and adhesion events occur at a high frequency during crude oil or *n*-hexadecane degradation of H9-3 strain cells. It is suggested that in nutrient rich medium (LB), oxygenase and adhesin are not needed by the H9-3 strain while these genes are translated to a greater extent in *n*-hexadecane or crude oil cultured cells. That is to say, the H9-3 strain prefers to exploit oxygenase and adhesin during the processes of crude oil or *n*-hexadecane degradation. The oxidation and adhesion events occur specifically during the process of crude oil or *n*-hexadecane consumption by bacteria.

In terms of adhesin and invasion, much is known about the adhesion and invasion of epithelial cells exploited by pathogenic bacteria, but little is known about alkane degradation. It was confirmed that the secretion of adhesin is beneficial to the uptake of insoluble alkanes through the high-expression of the G17 gene in the H9-3 strain. It was reported that *Acinetobacter calcoaceticus* strains can secrete emulsan which is an excellent bio-emulsifier for crude oil [33,34]. Because both of them have a hydrophile-lipophile ability, this research might prove that emulsan and adhesin (invasin) are products expressed from the same gene.

During the *n*-hexadecane degradation process, the expression of some genes in H9-3 cells, such as G5, G12, G15, G18, and G21, was down-regulated. This may be because the functions of these genes are not associated with the degradation of alkanes, for example, the G12, G15, and G21 genes, which are related to the benzoate 1,2-dioxygenase beta subunit and the benzoate membrane transport protein, while the G18 gene is related to bacteria ferritin-associated ferredoxin [35]. However, in the process of crude oil degradation, the expression of the G15 and G21 genes was not down-regulated, which may be due to the presence of not only alkanes but also a small amount of aromatic hydrocarbons in the composition of the crude oil.

## 5. Conclusions

In conclusion, a bacterial strain capable of degrading crude oil was isolated from crude oil-contaminated soil in the Northeastern part of China, and it was identified as *Acinetobacter pittii* H9-3. *A. pittii* H9-3 has the ability to grow in liquid medium with crude oil being the sole carbon and energy source. The H9-3 strain was able to degrade 36.8% of the initial 10 g·L${}^{-1}$ of crude oil. The H9-3 strain had preference for *n*-C10, *n*-C11, *i*-C14, *i*-C17, *i*-C34, *n*-C13, *n*-C14, *n*-C17, *n*-C32, and *i*-C13 rather than *n*-C16, *n*-C18–C22, *n*-C24–*n*-C31, and *n*-C36. This can be regarded as the specific fingerprint for crude oil degradation by the *Acinetobacter pittii* H9-3 strain and as the difference between *A. pittii* and *A. calcoaceticus*.

For *n*-hexadecane, when it was in the crude oil as one of the components, it could not be degraded by the H9-3 strain. However, when it was the sole carbon source, it could be degraded by the H9-3 strain.

In addition to crude oil, the H9-3 strain can utilize soybean or phenols as the sole source of carbon and energy. The order of preference for the carbon source of the H9-3 bacteria strain was shown to be soybean, followed by crude oil and phenols. The H9-3 strain tends to degrade crude oil rather than phenols, and this could be regarded as the difference between the *A. pittii* and *A. calcoaceticus* strains. *A. pittii* H9-3 showed a tolerance for α-naphthol and aniline at a concentration as high as 100 mg·L${}^{-1}$ and 1000 mg·L${}^{-1}$. The H9-3 strain showed no tolerance for methylbenzene (0.1%). Methylbenzene was neither utilized nor tolerated by strain H9-3.

Accumulation in pristane and 2,6,10-trimethyl-pentadecane could hinder a further increase in the degradation rate during the disposal of crude oil by the *A. pittii* H9-3 strain.

The difference in gene expression was significantly induced by diverse carbon sources. This paper revealed that oxidation and adhesion events occur at a high frequency during alkane or crude oil degradation by *Acinetobacter pittii* H9-3 strain cells. The research demonstrated the overexpression of genes for oxidation (G14) and adhesion (G17) that were employed exclusively to consume alkanes, while genes for bacteria ferritin-associated ferredoxin (G18) were down-regulated. At the same time, genes related to the benzoate 1,2-dioxygenase beta subunit (G15) and benzoate membrane transport protein (G21) which could act on aliphatic alkanes were down-regulated during the degradation process of *n*-hexadecane but not in crude oil.

## Figures and Tables

**Table 1:** Primer sequences for qRT-PCR.

Code	Primer Sequence	Length (bp)	Gene ID
P2	f-CGGCTTTTTGAGATTAGCATC-	188	*
	r-CGCAACCCTTTTCCTTATTTG-		
G5	f-CAGCCAATAAAGGTCGTAGCA	112	gi|325121063|gb|CP002177.1|:3263986-3265761
	r-CGGAAGTCAATAGCGTCTGTC		
G12	f-TGGGACGACGACGATAGATTA-	212	gi|325121063|gb|CP002177.1|:c510669-510157
	r-TTCCAGTTAAAGCGAACAGTGA-		
G14	f-AAGAAACTGCTGCTGAACACG-	233	gi|325121063|gb|CP002177.1|:210587-211186
	r-CCCGTTGGGTTGAATACCTA-		
G15	f-ACCCTATCTGACGCAGCCTAT-	216	gi|325121063|gb|CP002177.1|:c510104-509088
	r-TTGAATCTGGAATACCGCATC-		
G17	f-TGGAGATGAAGTTGAGGCAAT-	129	gi|325121063|gb|CP002177.1|:c2325664-2318453
	r-GCTGGTGTGCTGTCGTTAGTT-		
G18	f-CGCTGAAAGCTATCGTGAAAT-	108	gi|325121063|gb|CP002177.1|:2835719-2835901
	r-GCGATTTCTGCTAATTCTTCG-		
G21	f-GCCAGCCAAACCCATTATTAC-	242	gi|325121063|gb|CP002177.1|:c508226-507033
	r-CTGCCACCAACTCTTTAGGAA-		

* Internal reference gene [30].

**Table 2:** Growth of the H9-3 strain in several carbon sources for 7 days.

Carbon Source	(The Values of Optical Density at 600 nm)		
	3 days	5 days	7 days
Crude oil	0.30	0.25	0.28
Soybean oil	0.56	1.55	2.88
Phenols	0.11	0.19	0.24
α-Naphthol	0.06	ND	ND
Aniline	0.02	ND	ND
Methylbenzene	0	ND	ND

ND, not determined; 0, no growth.
